# Rapid Solidification of Plant Latices from *Campanula glomerata* Driven by a Sudden Decrease in Hydrostatic Pressure

**DOI:** 10.3390/plants14050798

**Published:** 2025-03-04

**Authors:** Arne Langhoff, Astrid Peschel, Christian Leppin, Sebastian Kruppert, Thomas Speck, Diethelm Johannsmann

**Affiliations:** 1 Institute of Physical Chemistry, Clausthal University of Technology, Arnold-Sommerfeld-Str. 4, 38678 Clausthal-Zellerfeld, Germanyastrid.peschel@tu-clausthal.de (A.P.);; 2 Analytical Chemistry II—Shape-Dependent Electrochemistry, Ruhr-University Bochum, Universitätsstr. 150, 44801 Bochum, Germany; 3 Plant Biomechanics Group and Botanic Garden, University of Freiburg, Schänzlestr. 1,79104 Freiburg, Germany

**Keywords:** self-healing, plant latices, QCM-D, solidification, liquid–liquid phase separation

## Abstract

By monitoring the solidification of droplets of plant latices with a fast quartz crystal microbalance with dissipation monitoring (QCM-D), droplets from *Campanula glomerata* were found to solidify much faster than droplets from *Euphorbia characias* and also faster than droplets from all technical latices tested. A similar conclusion was drawn from optical videos, where the plants were injured and the milky fluid was stretched (sometimes forming fibers) after the cut. Rapid solidification cannot be explained with physical drying because physical drying is transport-limited and therefore is inherently slow. It can, however, be explained with coagulation being triggered by a sudden decrease in hydrostatic pressure. A mechanism based on a pressure drop is corroborated by optical videos of both plants being injured under water. While the liquid exuded by *E. characias* keeps streaming away, the liquid exuded by *C. glomerata* quickly forms a plug even under water. Presumably, the pressure drop causes an influx of serum into the laticifers. The serum, in turn, triggers a transition from a liquid–liquid phase separated state (an LLPS state) of a resin and hardener to a single-phase state. QCM measurements, optical videos, and cryo-SEM images suggest that LLPS plays a role in the solidification of *C. glomerata*.

## 1. Introduction

### 1.1. Fast Solidification in Nature and in the Technical World

Between 8 and 10% of the flowering plants in nature exude a latex when injured [1,2]. The term “latex” in this context denotes a milky fluid, which solidifies after exudation, thereby protecting the plant [2,3,4]. Protection encompasses “self-sealing” and “self-healing” [3], where self-sealing precedes self-healing. Even a liquid material may prevent the entry of toxins into the plant (self-sealing), while self-healing requires some degree of solidification. Evidently, fast self-healing amounts to an evolutionary advantage.

We briefly digress on the solidification of technical latices, also termed polymer dispersions. Polymer dispersions amounted to a market volume of more than USD 10 billion in 2023 [5]. Important products are coatings [6,7], adhesives [8,9], and rubber gloves [10]. Film formation from water-borne polymer dispersions has been studied in considerable depth [11]. The mechanisms of solidification can be roughly grouped as physical drying [12], chemical crosslinking [13], polymer interdiffusion [14], and wet sintering [10]. Physical drying in air can induce deformation and merging of particles with a glass temperature close to the drying temperature because the deformation in this case is driven by a large negative capillary pressure originating from the concave menisci at the film–air interface [12]. Chemical crosslinking is widely employed to achieve toughness [13,15]. The diffusion of polymer chains across the interparticle boundaries—often at elevated temperature and over prolonged time—also contributes to toughness [16]. Wet sintering is employed in a process called coagulation dipping [17]. The object to be covered (the “former”, shaped as a hand in the production of rubber gloves) is first coated with a coagulant (a layer of a calcium salt) and then dipped into the latex dispersion. After the electrostatic repulsion between spheres has been overcome by Ca^2+^ bridges, particles coalesce onto the former, driven by the van der Waals attraction and the interfacial tension between the polymer and the liquid. Coagulation dipping requires soft materials such as rubbers because the stress in wet sintering cannot deform stiff spheres.

Both physical drying and drying-induced crosslinking take tens of seconds, at least, because they require diffusive transport of water or reaction inhibitors from the bulk to the film–air interface. If crosslinking is induced by oxygen, the oxygen again must diffuse from the film–air interface into the bulk. At room temperature and with a film thickness above 300 µm, the drying time is closer to a few minutes than to a few seconds. This estimate follows from the diffusion time scale, τ_diff_, being given as τ_diff_ = *L*^2^/*D* with *L* as the characteristic length (*L* > 300 µm) and *D* as the diffusivity (*D* < 10^−5^ cm^2^/s). Drying time is of critical importance when laminating aluminum foils while these run through an oven at high speed. This process has received renewed attention in the context of electrode formation for lithium ion batteries [18]. These consist of special coatings on an aluminum backing, where the latter is the current collector. Drying time translates into path length inside the oven and thereby into cost. The rate limiting step can be diffusion inside the film, but also the rate of evaporation [19].

This work is concerned with a solidification process—namely the self-healing of the latex exuded by *Campanula glomerata*—which is too fast to be caused by physical drying. As is well known from the technical world, solidification can also be triggered by mixing processes on the local scale. These require a mechanism, by which the reagents (often called “resin” and “hardener”) are maintained separate prior to solidification and are mixed once solidification is supposed to set in. Microcompartments achieving separation may be capsules with a wall, which break when a suitable stimulus is applied. In the case of the “self-healing resins”, the stimulus is a crack that intersects the capsule containing the hardener [20]. Other stimuli are temperature, biodegradation [21], or stress [22]. Similar processes are exploited in the context of “smart coatings”, which are reviewed in Ref. [23], and in active packaging, which are reviewed in Ref. [24]. In principle, physical drying can also be a stimulus, but physical drying is slow and therefore cannot explain rapid solidification.

Another example, where a hardener is released from a capsule, is the rubber tree (*Hevea brasiliensis*). The hardener in *H. brasiliensis* is the protein hevein, contained in capsules named “lutoids” [25]. Pakianathan et al. noticed as early as 1966 that lutoids may break following an osmotic shock [26]. D’Auzac et al. propose that the osmotic shock is the consequence of an influx of serum into the capillaries containing the lutoids (the “laticifers”) [27]. This influx is the consequence of a sudden decrease in pressure inside the capillaries, as expected upon exudation. D’Auzac et al. do not emphasize that the process can be fast and it actually is not particularly fast in the case of the rubber tree. It can be fast though because a pressure drop propagates through the sample with the speed of sound and because all follow-up processes happen on a local scale. The d’Auzac model is compatible with fast solidification.

### 1.2. A Possible Role of Liquid–Liquid Phase Separation (LLPS) in Solidification

The problem with the d’Auzac model applied to *C. glomerata* is that images of the exudate acquired with cryo-SEM did not show any evidence of capsules [28]. The d’Auzac model requires capsules and it is difficult to understand why such capsules should not at all be visible to cryo-SEM.

Another conceivable mixing process on the local scale is linked to a transition from liquid–liquid phase separation (LLPS) to a single-phase state, triggered by pressure [29]. LLPS is rather common in biological systems, often in the form of condensed globules of proteins. LLPS can also amount to complex formation between two sets of polyelectrolytes with opposite charge. The name “coacervation” is often used in the context of polyelectrolytes, but is also applied to biocondensates. LLPS and its biological implications have been reviewed in-depth by Cinar et al. [29]. Liu et al. describe the role of LLPS in fibrillar networks [30]. Rose et al. report an experiment, where coacervation was used to turn a fluid latex dispersion into a gel without drying [31]. The authors call the mechanism “controlled ionic coacervation” (CIC).

Among the parameters modulating LLPS are protein concentration, ionic strength, osmolyte content, and pressure. Pressure affects the intermolecular interactions and may thereby induce a transition from the two-phase state to the single-phase state, at least in principle. However, a more detailed look at the examples from Ref. [29] raises doubt on pressure being the direct cause of such a transition in *C. glomerata*. The pressures involved are in the range of hundreds of bars (see Figure 6 in Ref. [29]), while the pressure inside the laticifers is closer to a few bars. More likely, the transition is mediated by an influx of serum into the laticifers, where the latter is caused by the pressure drop. The influx changes the concentrations of ions and osmolytes inside the laticifers. A similar, indirect path is claimed in the case of the rubber tree. The osmotic shock in the d’Auzac model is caused by an influx of serum, as opposed to altered hydrostatic pressure.

### 1.3. Previous Work on C. glomerata

Fast solidification in the case of *C. glomerata* and the pressure-drop mechanism playing a role in the latex of solidification from *Ficus benjamina* have been discussed in different publications by the Freiburg group [32]. Bauer et al. describe a setup, which allowed them to damage plants under pressure [1]. Latex solidification was very delayed in *Hevea brasiliensis* and *Ficus benjamina* when the plant was injured at a pressure above 8 bars. Because coagulation in the case of *C. glomerata* was too fast for these experiments, the Freiburg group conducted a second simple test [32]. They cut the stem of *C. glomerata* and repeatedly pulled on the white fluid with the same scalpel, which was used to make the cut. Immediately after the cut (~1 s), the latex was in a liquid state. The second or third pull (2–5 s later) produced threads between the stem and the tip of the scalpel. At this time, an elastic skin had formed.

The Freiburg group undertook a third experiment [33], which motivates the work reported below. Solidification of *F. benjamina* and *E. characias* latex was monitored using nanorheometers in an initial oscillating mode followed by a stepped-shear viscosity test. Parallel measurements of the drying rate based on gravimetry allowed them to distinguish between evaporation effects in *Euphorbia* spp. and chemical reactions as found in *F. benjamina*. As with the high-pressure measurements, solidification in the case of *Campanula* spp. was too fast to be followed with this setup. We expand on these studies using a fast rheometer, which gives access to the drying of *C. glomerata*. More specifically, we employ a quartz crystal microbalance with dissipation monitoring (QCM-D, QCM, for short), which is described in Section 2 in more detail. The high data acquisition rate allows us to resolve the solidification kinetics. We further corroborate the explanation in terms of a pressure change with optical videos from plants being cut under water. These videos test whether solidification requires the presence of air.

## 2. Experimental Procedures

### 2.1. The QCM as an Instrument to Study Solidification

**Figure 1:**
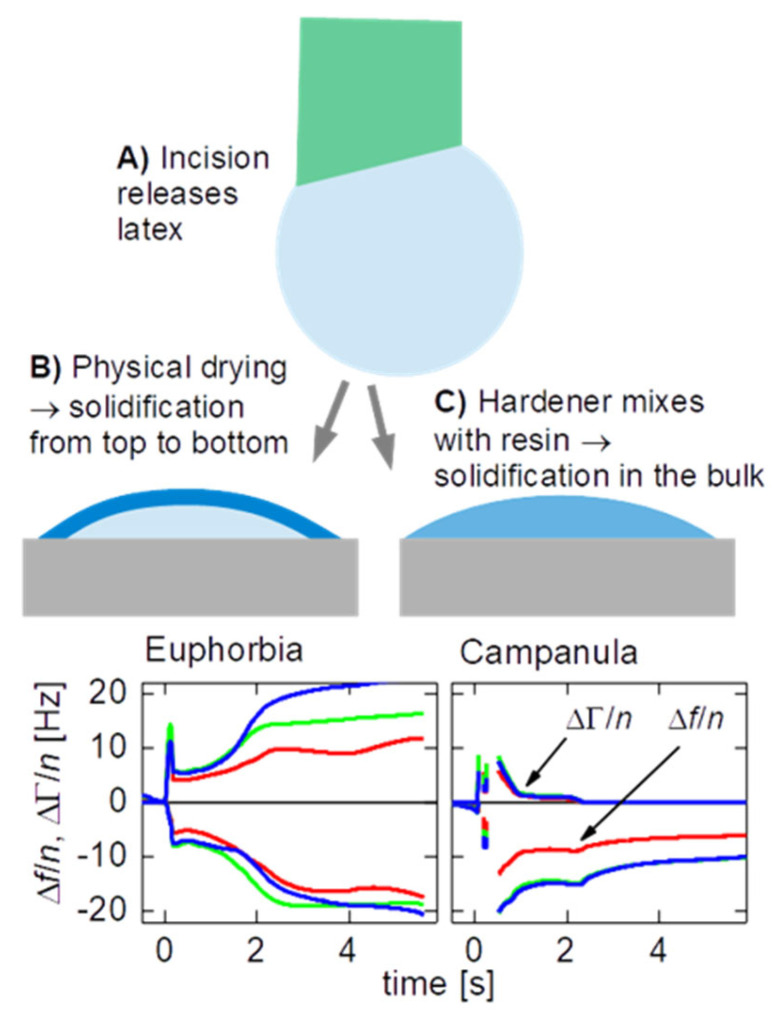
Sketch of the experimental protocol and two data traces. In the case of *C. glomerata*, the half bandwidth quickly decreases to zero, evidencing rapid solidification. (**A**): Latex is exuded from the stem. (**B**,**C**) Depending on whether drying is induced by the contact with air or by a change in hydrostatic pressure, solidification occurs as a skin first (**B**) or not (**C**). The colors in the diagrams indicate the overtones. The overtones at 15, 25, and 35 MHz are shown in red, green and blue.

A QCM consists of a piezoelectric plate, which is electrically excited to thickness shear vibrations with frequencies in the MHz range. Contact between the resonator and a sample shifts the resonance frequencies and the resonance bandwidths. When the sample is a liquid droplet, the kinetics of drying and/or solidification can be inferred from the evolution of frequency and bandwidth with time. Because the shear wave emanates from the resonator surface, the QCM is rather insensitive to the formation of a skin at the droplet–air interface. It mostly probes the bulk (Figure 1C). A few microliters of sample suffice (even less, see Ref. [34]), which is among the advantages of the QCM-D, compared to other rheometers. Also, droplets can be deposited onto the resonator plate quickly. Cutting the stem of the plant and collecting the liquid takes more time than placing the sample onto the resonator plate. Other types of microrheometers do not allow for fast sample entry in the same way [35].

The Clausthal group has recently demonstrated a QCM-D with a much improved rate of data acquisition (Section 2.2). In all respects other than time resolution, which is 10 milliseconds, the work reported below follows the lines of Bauer et al. [33]. Data acquisition is started, a droplet of latex is deposited on the resonator surface, and the droplet is allowed to solidify (Figure 1). It takes some practice to cut the stems open and to then quickly place the droplet on the resonator surface (with a delay of about 10 s). Otherwise, the procedures are rather simple.

### 2.2. A Fast Quartz Crystal Microbalance Interrogated by a Multifrequency Lockin Amplifier

On the technical side, we exploit the options opened up by a fast, multi-overtone QCM-D, which makes use of a multifrequency lockin amplifier (MLA, supplied by Intermodulation Products SE, Stockholm, Sweden) [36]. The instrument determines shifts in frequency, Δ*f*, and in half bandwidth, ΔΓ, on up to four overtones simultaneously, where overtones usually are labeled by their overtone order *n*. Overtones at *n* = 3, 5, and 7 with frequencies of 15, 25, and 35 MHz were interrogated here. In the “comb mode”, the MLA sends out up to 32 sine waves, arranged as one or a few combs in frequency, covering one resonance or the three resonances at 15, 25, and 35 MHz. The MLA collects the corresponding currents at these 32 frequencies, thereby determining the electrical admittance of the device under test at these frequencies. Plots of the complex admittance versus frequency show resonance curves, which can be fitted with “phase-shifted Lorentzians” [37]. The fit derives the resonance frequency and the resonance bandwidth.

Transformed to the time domain, a frequency comb amounts to a series of pulses spaced in time by δ*t*_comb_ = 1/δ*f*_comb_ with δ*f*_comb_ being the gap between neighboring frequencies. The gap therefore sets the time resolution of the measurement. δ*f*_comb_ must be less than the bandwidth of the resonance because the comb will otherwise miss the resonance. With a resonance bandwidth of a few hundred Hz, δ*f*_comb_ can be 100 Hz, which puts the time resolution to 10 ms.

For planar films, one might compare the values of Δ*f*/*n* between overtones, which would lead to a statement on the film’s softness. For droplets, the poorly controlled geometry prevents such a quantitative analysis [34,38]. Solidification per se, however, is easily inferred from the kinetics of the bandwidth. The bandwidth is proportional to the energy dissipated per unit time in the sample. Soft samples dissipate energy, while rigid samples do so to a lesser extent. There is a slight caveat insofar, as coupled resonances can complicate the picture [39]. These occur when the sample itself has an acoustic eigenfrequency close to the resonance frequency of the QCM. Droplets should be as small as possible for that reason. However, the droplets were never thinner than a few 100 µm. At this size, physical drying requires a few minutes, at least.

## 3. Results

### 3.1. QCM Experiments

All plants were purchased in a local store. In the main text, we report experiments on two species, which are *C. glomerata* (a flower, English names are “clustered bellflower” or “dane’s blood”) and *E. characias* silver swan (English name: “Mediterranean or Albanian spurge”). The supporting information also shows QCM experiments on *Ficus benjamina* and two technical latexes. While there was some variability, systematic trends or differences between plant latices and technical latices were not seen. *C. glomerata* was the one exception. It solidified much faster than all other samples studied.

**Figure 2:**
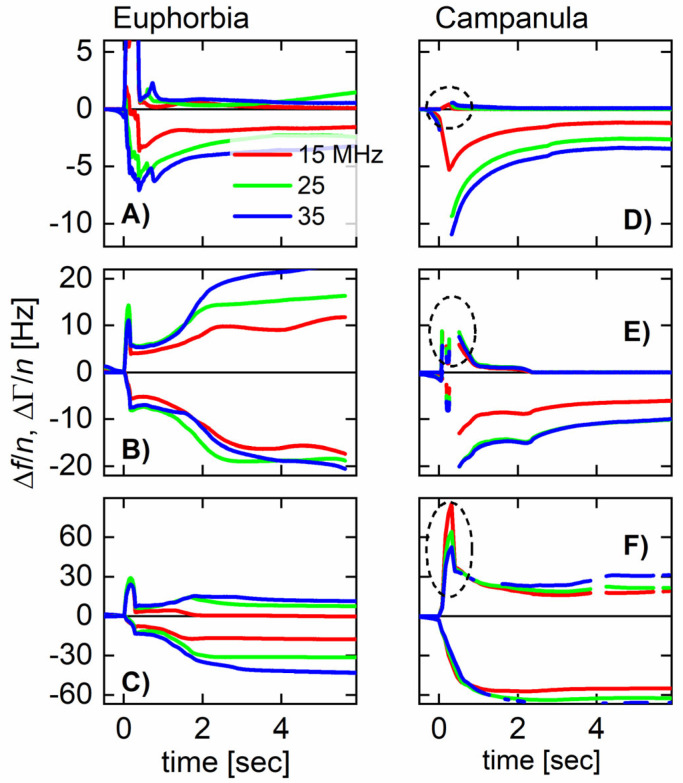
Time traces of the overtone-normalized shifts in the frequency, Δ*f*/*n* (always Δ*f*/*n* < 0), and the half bandwidth, ΔΓ/*n* (always ΔΓ/*n* > 0). In the case of *C. glomerata* (**D**–**F**), the half bandwidth decreases more quickly than in the case of *E. characias* (**A**–**C**). The sharp peaks in ΔΓ/*n* are indicated with dashed ellipses. *t* = 0 is the time when the droplet is deposited on the resonator. The incision to the plant had been made a few seconds earlier.

Figure 2 shows three examples (for each *E. characias* and *C. glomerata*) of how the shifts in overtone-normalized frequency, Δ*f*/*n*, and overtone-normalized half bandwidth, ΔΓ/*n*, evolve with time. Data from three overtones at 15, 25, and 35 MHz are shown. The overtone-normalized frequency shifts never agreed between overtones. A rigid layer would induce an overtone-normalized frequency shift Δ*f*/*n* independent of overtone order. The samples studied here do not appear as rigid to the QCM. In panels D and E (*C. glomerata*), ΔΓ/*n* rapidly decreases to zero. These droplets do not dissipate energy after about a second. It must be noted that it took about 10 s to cut the stem and place the droplet on the resonator. Energy dissipation stopped at a time of less than 12 s after the cut was made.

In panel F (also *C. glomerata*), there is a fast decrease, which, however, does not lead to zero. Judging from the frequency shift (and also from visual observation), this droplet is larger than the droplets from panels D and E. In the presence of a small viscous component of the sample’s compliance, ΔΓ increases in proportion to the cube of the sample’s thickness (Equation (44) in Ref. [37]). Because this droplet is thicker than the two others, ΔΓ/*n* levels off to a finite value after solidification.

The phenomenology is very different in panels A–C (*E. characias*). In these cases, ΔΓ/*n* stays large over the duration of these experiments. ΔΓ/*n* does eventually drop after about a minute.

### 3.2. Video Microscopy on Samples Injured in Air and Under Water

**Figure 3:**
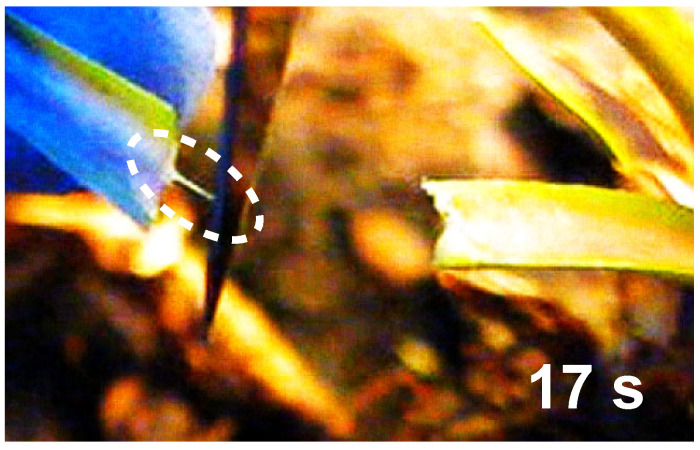
A frame from an optical video, showing an incision into the stem of *C. glomerata*, where 17 s after the cut was made, the white milky material that exuded from the cut forms a thread, when pulled on with the scalpel. The thread is indicated with a dashed ellipse.

**Figure 4:**
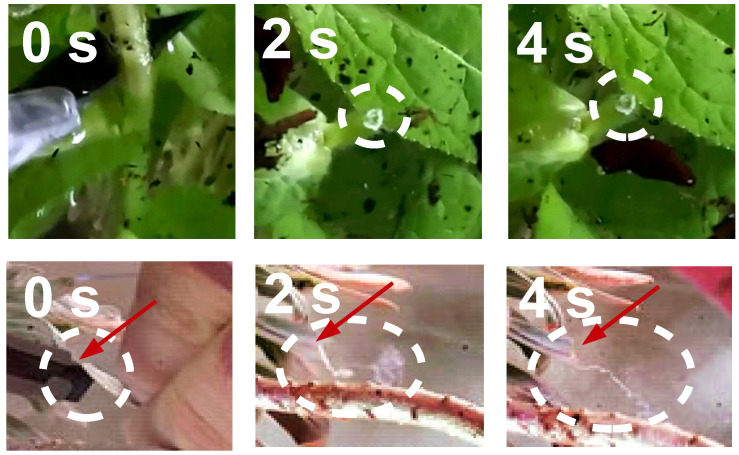
Frames from optical videos of experiments under water. After the plants have been injured, they exudate latex. In the case of *C. glomerata*, the latex immediately forms a plug. The white structure in the center of the dashed circle at the top is the plug formed by the latex. In the case of *E. characias*, the latex streams away, forming a filamentous structure (bottom, indicated with a red arrow).

Rapid solidification in the case of *C. glomerata* is impressively evidenced by simply cutting the stem of the plant with a scalpel and pulling on the liquid (Figure 3). An elastic thread soon forms in the case of *C. glomerata*, while the thread appears much later in the case of *E. characias*. These experiments confirm the findings by Bold [32]. The video traces, however, are less conclusive than the QCM experiments with regard to the mechanism of solidification because what appears to be a thread might consist of a liquid cylinder surrounded by skin. This skin may form quickly because both the evaporation of water and the entry of oxygen is fast close to the surface. There is a similar experiment that avoids this ambiguity. When the incision is made under water (Figure 4), latex from *C. glomerata* immediately forms a plug. Clearly, neither air nor physical drying are required for solidification. Latex from *E. characias*, in contrast, keeps streaming away over the entire duration of the experiment, which was about one minute.

The supporting information contains links to web addresses, where the videos mentioned in Figure 3 and Figure 4 can be viewed. It also contains a link to a video, where a cut was made to *Ficus benjamina* under water. As in the case of *E. characias*, the latex stays in liquid form and streams away.

## 4. Discussion

Rapid solidification is evidenced by optical videos and the QCM. The first set of arguments supporting the pressure-drop hypothesis is based on kinetics alone. All other mechanisms require transport of water to the droplet surface or transport of oxygen from the surface into the bulk. If the diffusion length is larger than 300 µm, diffusive transport requires a minute, at least. The QCM as a tool to quantify the solidification kinetics has two advantages. First, the data are more quantitative than what can be learned from the inspection of optical videos. Admittedly, there was some variability in how fast the droplets were transferred from the stem of the plant to the resonator surface (≈10 s). Only after this transfer is complete does the QCM experiment start. Still, the QCM shows that solidification occurs within seconds. A second advantage with regard to this problem is that the QCM probes the droplet from below. Should a skin form at the droplet–air interface, the QCM will be insensitive to the skin as illustrated in the sketches in Figure 1B,C. A skin might form within seconds even when the process is rate-limited by diffusion because the diffusion path is short at the surface.

With regard to excluding physical drying as the cause for crosslinking, the pulling experiment in air is less conclusive than the QCM experiment. The experiment, where plants are injured under water, is conclusive because it avoids exposure to air. These arguments do not exclude some influence of pressure in the case *E. characias* and the other plants studied (such as *F. benjamina*, see the Supplementary Materials). Following the lines of the d’Auzac model, we propose the influx of serum into laticifers as the key intermediate step, triggering solidification. The mostly plausible mechanism for mixing is a transition from a liquid–liquid two-phase state to a single-phase state. Following this hypothesis, an influx of serum into the laticifers after injury is rather common. The difference between *C. glomerata* and other plants is in whether or not the influx causes gelation.

The authors are not aware of cases where similar arguments would apply to the drying of synthetic latices. These do not usually contain capsules (which would break) or globules (which would dissolve) following a pressure drop. All polymer dispersions studied here solidified slowly (Section S1 in the Supplementary Materials).

This leaves the question of whether there are ways to take advantage from fast solidification in a technical context. This might concern the drying process or responsivity in smart coatings [23] and active packaging [24]. A pressure drop is not easily realized in such settings, but a temperature jump within a few seconds or even less might be, at least for sufficiently thin films. Conceptionally, one could borrow ideas from controlled drug release and also underwater adhesion building on triggered coacervation [40,41]. For instance, drug release was triggered by hyperthermia in ref. [42]. LLPS can be modulated by temperature easily [43]. Materials with an upper critical solution temperature (UCST) are needed because the temperature jump will always amount to heating and because the single-phase state must be the high-temperature state. In water, UCST behavior is less common than LCST behavior (that is, phase segregation at the higher temperature). UCST behavior combined with LLPS was reported in Kim et al. [44]. In the context of underwater adhesion, the solid state often is the coacervate state. Coacervation might be achieved with proteins or with polyelectrolyte complexes. Polyelectrolyte complexes presumably are more cost-efficient, which is important for packaging due to the large volumes involved. Such methods would have to be evaluated against UV curing [45], which has similar advantages and is carried out in a similar way.

## 5. Conclusions and Implications

Using a fast QCM-D, it was shown that latex droplets from *C. glomerata* solidify much faster than droplets from other plants and also droplets containing technical latices. In parallel experiments, plants were injured under water. The latex from *C. glomerata* formed a plug, while the latex from *E. characias* and *F. benjamina* did not. The solidification occurred in the bulk fluid with no influence of physical drying.

The fast kinetics suggest that solidification in the former case is triggered by a sudden decrease in pressure inside the laticifers, followed by an influx of serum into the laticifers, leading to the local mixing between a resin and a hardener. Presumably, mixing is caused by a transition from a liquid–liquid phase separated state to a single-phase state. At this point, species of the genus *Campanula* are the only plants displaying rapid solidification of this kind, known to us. Similar mechanisms are occasionally put to use in the health care industry. They may also find use in smart coatings and active packaging.

## Data Availability

The original contributions presented in this study are included in the article/Supplementary Materials. Further inquiries can be directed to the corresponding author.

## Supplementary Materials

The following supporting information can be downloaded at: https://www.mdpi.com/article/10.3390/plants14050798/s1. The supporting information shows more examples, where the drying kinetics of droplets were acquired with a QCM (*Ficus benjamina* and two technical latexes). It shows a screen shot from a video similar to the bottom section of Figure 4 but taken using *F. benjamina*. It provides web addresses, from which the videos can be downloaded.
