# Supplementary material for: Rapid Solidification of Plant Latices from Campanula glomerata Driven by a Sudden Decrease in Hydrostatic Pressure
Source: Plants (Basel). 2025 Mar 4;14(5):798. doi: 10.3390/plants14050798 (PMC11902487; doi:10.3390/plants14050798)
Supplement: Supplementary file 1 [file plants-14-00798-s001.zip › plants-3395876-supplementary.pdf]

Rapid Solidification of Plant Latices from Campanula glomerata Driven by a Sudden Decrease in Hydrostatic Pressure

Arne Langhoff,[1] *Astrid Peschel,*[1] *Christian Leppin,*[1,3]
Sebastian Kruppert,[2] *Thomas Speck,*[2] *and Diethelm Johannsmann*[1*]

[1]Institute of Physical Chemistry, Clausthal University of Technology, Arnold-Sommerfeld-Str. 4, 38678 Clausthal-Zellerfeld, Germany

[2]Plant Biomechanics Group and Botanic Garden, University of Freiburg, Schänzlestr. 1, 79104 Freiburg, Germany

[3]Ruhr-University Bochum, Analytical Chemistry II - Shape-dependent Electrochemistry, Universitätsstr. 150, D-44801 Bochum, Germany

[*]Correspondence: johannsmann@pc.tu-clausthal.de
ORCID ID: 0000-0002-8873-1742

Supporting Materials

Figure S1…….More data showing the solidification of latex droplets as determined with a QCM....... 2

Figure S2........A screenshot from a video, where Ficus benjamina was injured under water................ 3

Figure S3 ..... Web addresses, where the videos underlying Figs. 3.2 and 3.3 from the main text and the figure from section 2 can be viewed... 3

Figure S1: More data showing the solidification of latex droplets as determined with a QCM

The main text focused on two plants, which were *Campanula glomerata* and *Euphorbia characias.* The figure below shows similar data obtained from *Ficus benjamina* and from two technical latexes. In these cases, drying invariably took longer than a few seconds.

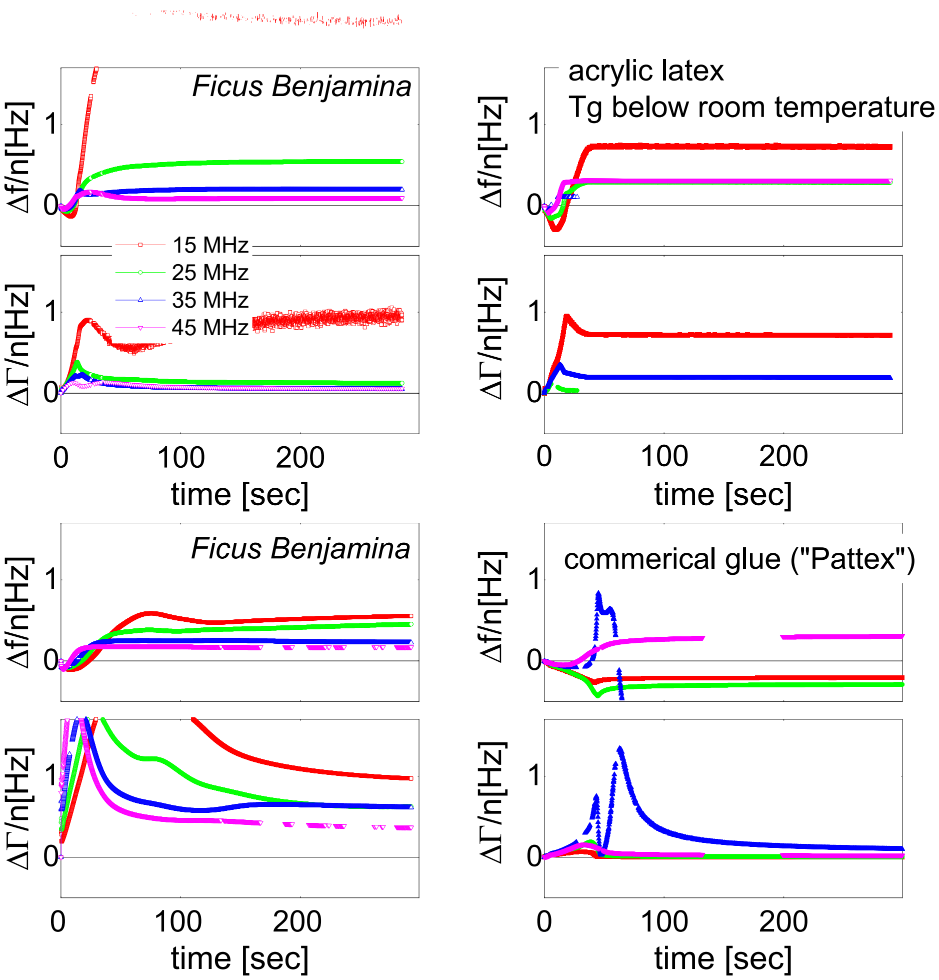

Figure S2 A screenshot from a video, where Ficus benjamina was injured under water

The figure below is an analog to the bottom part from Fig. 4 in the main text, but the plant under study is *Ficus benjamina*. Similar to what is shown for *Euphorbia characias* in the main text, the liquid keeps streaming away.

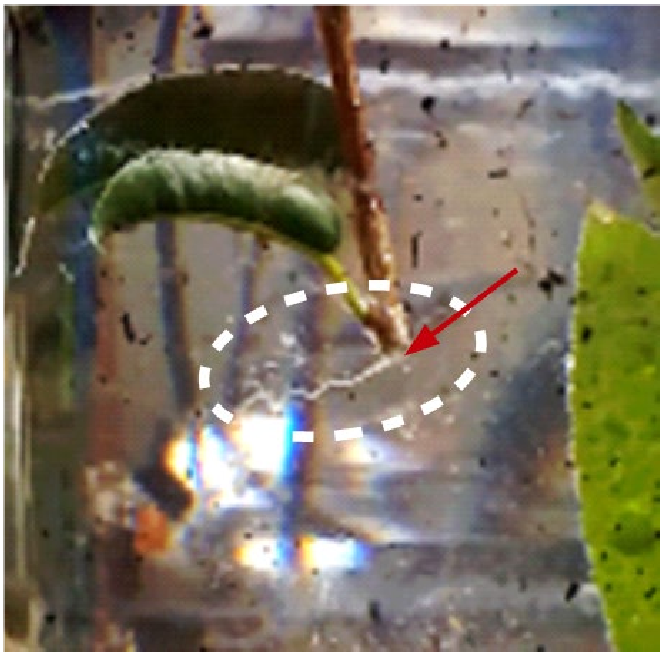

Figure S3. Web addresses, where the videos underlying Figs. 3.2 and 3.3 from the main text and the figure from Section 2 can be viewed

The videos are available for download from

https://www.pc.tu-clausthal.de/fileadmin/Sites/PC/Videos/Campanula_glomerata_cut_in_air.mp4
https://www.pc.tu-clausthal.de/fileadmin/Sites/PC/Videos/Campanula_glomerata_cut_in_water.mp4
https://www.pc.tu-clausthal.de/fileadmin/Sites/PC/Videos/Euphorbia_characias_cut_in_water.mp4
https://www.pc.tu-clausthal.de/fileadmin/Sites/PC/Videos/Ficus_benjamina_cut_in_water.mp4
